# Performance Comparison between Polyvinylidene Fluoride and Polytetrafluoroethylene Hollow Fiber Membranes for Direct Contact Membrane Distillation

**DOI:** 10.3390/membranes9040052

**Published:** 2019-04-11

**Authors:** Frank Y. C. Huang, Allie Arning

**Affiliations:** Department of Civil and Environmental Engineering, New Mexico Tech, Socorro, NM 87801, USA; allie.arning@student.nmt.edu

**Keywords:** DCMD, membrane distillation, desalination, hollow fiber membrane, PVDF, PTFE, water flux, field performance, pilot plant, geothermal, greenhouse

## Abstract

Increasing water demand coupled with projected climate change puts the Southwestern United States at the highest risk of water sustainability by 2050. Membrane distillation offers a unique opportunity to utilize the substantial, but largely untapped geothermal brackish groundwater for desalination to lessen the stress. Two types of hydrophobic, microporous hollow fiber membranes (HFMs), including polytetrafluoroethylene (PTFE) and polyvinylidene fluoride (PVDF), were evaluated for their effectiveness in direct contact membrane distillation (DCMD). Water flux and salt rejection were measured as a function of module packing density and length in lab-scale systems. The PVDF HFMs generally exhibited higher water flux than the PTFE HFMs possibly due to thinner membrane wall and higher porosity. As the packing density or module length increased, water flux declined. The water production rate per module, however, increased due to the larger membrane surface area. A pilot-scale DCMD system was deployed to the 2nd largest geothermally-heated greenhouse in the United States for field testing over a duration of about 22 days. The results demonstrated the robustness of the DCMD system in the face of environmental fluctuation at the facility.

## 1. Introduction

Traditionally, the bulk of the United States (U.S.) water demand has been satisfied by a combination of surface water and fresh groundwater. However, as climate and land use change, resources deplete, and population grows, these sources are not expected to suffice; thus, unconventional water resources are needed to prevent serious socio-economic consequences. Brackish groundwater represents a substantial, but largely untapped resource [1]. A conservative low estimate for the volume of extractible brackish groundwater is about 3.7 trillion·m^3^ (3 billion acre-feet), more than 35 times the amount of fresh groundwater used in the U.S. during 2010 [2]. The utilization of this unconventional water resource however is very limited. Data from United States Geological Survey (USGS) indicates that an estimated 12.3 million·m^3^/day (10,000 acre-feet/day) of brackish groundwater was used in the U.S. in 2010, which constituted only 4% of total groundwater use [2]. In the Western U.S., substantial amounts of freshwater are used for agriculture activities. For example, in California, Arizona, and other western states where pressures on water availability are especially intense, up to 80% of total water use is consumed by irrigated agriculture [3]. The strain on the water supply is further exacerbated by the projected climate change, which may result in a 10–20% reduction of the groundwater recharge across southwestern aquifers by 2100 [2]. Therefore, it is especially urgent in this region to explore technologies that can utilize this vast quantity of unconventional resource as a supplement or replacement for freshwater supply. Reverse osmosis (RO) is a well-established technology for brackish water desalination. However, it is relatively energy-intensive, at times requires higher levels of pre-treatment to reduce membrane fouling, and has limited options for in-land concentrate management. For the western states of the U.S. where untapped brackish groundwater and geothermal resources are abundant, membrane distillation (MD) offers a unique opportunity to desalinate geothermal brackish groundwater with innate heat to supplement freshwater demand. In contrast to RO, MD is not a pressure-driven process and relies mainly on the vapor pressure gradient across hydrophobic membranes to drive the production of distilled water. Membrane distillation is especially attractive to horticulture and aquaculture operations that utilize geothermal brackish water as heating sources and where the demand of quality water for irrigation or cultivation is high.

DCMD is the simplest and the most commonly used configuration of MD for water desalination [4,5]. In DCMD, both the hot brackish feed and cold distilled permeate are in direct contact with a micro-porous membrane. Liquid water is prevented from wetting the membrane pores by controlling the pore size and hydrophobicity of the membrane. Water vapor generated from the hot feed is transported across the membrane via diffusion induced by the trans-membrane vapor pressure gradient, and condenses on the cold permeate side. High hydrophobicity is one of the key parameters in polymer selection to prevent pore wetting and subsequent performance deterioration in desalination. Polymers commonly used as membrane materials in DCMD include polyvinylidene fluoride (PVDF), polypropylene (PP), and polytetrafluoroethylene (PTFE). PTFE and PP exhibit higher hydrophobicity than PVDF; however, most researchers use PVDF to fabricate DCMD membranes due to its solubility in common solvents [5]. PTFE and PP, on the other hand, will have to be thermally extruded to make MD membranes. Many lab-scale DCMD studies have been conducted to demonstrate the ability of PVDF-based flat-sheet or hollow-fiber membranes for water desalination; however, limited data is available for their long-term performance in the field [6,7,8,9,10,11,12,13]. Gryta and Barancewicz evaluated the performance of PVDF capillary membranes with sponge-like or mixed sponge-/finger-like structures for lab-scale DCMD [14]. Significant decline (~60%) of water flux was observed for these systems after 1000 h of operation. The decline in water flux was attributed to progressive wetting of the pores. Pore wetting can significantly compromise the water flux and salt rejection of a DCMD system, leading to system failure. Inclusion of nanoparticles, such as PTFE, TiO_2_, SiO_2_, and carbon nanotube, and applications of extrinsic coatings with low surface energy have been investigated by researchers to increase the surface hydrophobicity of PVDF membranes, thereby reducing pore wetting [15,16,17,18,19,20,21,22,23,24,25,26]. These modifications, however, often suffer from complex manufacturing procedures, loss of the coatings in long-term operation, and potential health impact to human and the environment [27,28]. Compared to PVDF, PTFE possesses higher material hydrophobicity and lower chemical reactivity for applications in DCMD. Its performance reported in the literature, however, is almost exclusively for flat-sheet PTFE membranes; seldom for hollow fiber membranes (HFMs) [29,30,31,32,33,34,35,36,37,38]. Relative to flat sheet, the hollow-fiber configuration provides higher specific surface areas for membrane modules (e.g., HFM modules: up to 9000 m^2^/m^3^ versus spiral-wound modules: 1200 m^2^/m^3^), thus resulting in a higher productivity [39]. The hollow-fiber configuration is also self-supporting mechanically and therefore provides better flexibility during module fabrication and system operation [40]. This paper provides the much-needed performance data of PTFE HFMs for performance comparison. Preliminary field data collected for PVDF-based membrane modules at Masson Farms, the 2nd largest geothermally heated commercial greenhouse in the U.S., is also presented. The implication for system design and operation in the field is discussed.

## 2. Materials and Methods

### 2.1. Membrane Fabrication and Characterization

The PVDF hollow fiber membranes were fabricated using a dry jet-wet spinning process described in details elsewhere [13]. After spinning, the nascent fibers were submerged in de-ionized water for 24 h and subsequently freeze-dried to complete post-treatment before usage. The PTFE hollow fiber membranes fabricated using a melt-extrusion process were acquired from Markel Corporation (Plymouth Meeting, Montgomery County, PA, USA).

Morphologies of the hollow fiber membranes were characterized using scanning electron microscopy, SEM (Hitachi S3200N, Chiyoda-ku, Tokyo, Japan). Pore sizes and distributions were determined using capillary flow porometry (Quantachrome 3G zh, Boynton Beach, FL, USA). Membrane porosities were estimated using a gravimetric method. Mechanical properties of the fibers were measured at 20 °C using a tensile tester (Mark-10 ESM303, Copiague, NY, USA) equipped with a 100 N digital force gauge (Mark-10 Model M5-20, Copiague, NY, USA). Detailed methodologies for characterization were described in prior publications [13,41].

### 2.2. Module Fabrication

Individual fibers were hand sorted for defects before being fitted inside polycarbonate tubes with an inner diameter of 0.953 cm to achieve fiber packing densities up to 50%. Polycarbonate was chosen for its high thermal resistance and good optical clarity. The packing density of the hollow fiber membrane modules was calculated using Equation (1).

(1) $$Packing\;Density\;\left(\%\right)=\frac{A_{f}}{A_{m}\;}\times \;100\%$$

where $A_{f}$ is the total cross-sectional area of the fibers in a module and $A_{m}$ is the maximum packable cross-sectional area of the module determined by the mathematical software program Packomania [42]. Epoxy resin (Devcon Corporation) was then used to pot each module. The epoxy was cured at room temperature for 24 h followed by an additional 2–3 h at 50 °C. Prior to use, a section of the cured epoxy was removed from each end of the module to expose the lumen side of the hollow fiber membranes. Two types of membrane modules were prepared for testing with an effective fiber length of 9 cm and 24 cm, respectively. Each module underwent a QA/QC procedure in the lab after fabrication to check for module integrity before usage.

### 2.3. Experimental Setup for Lab-Scale Testing

**Figure 1:**
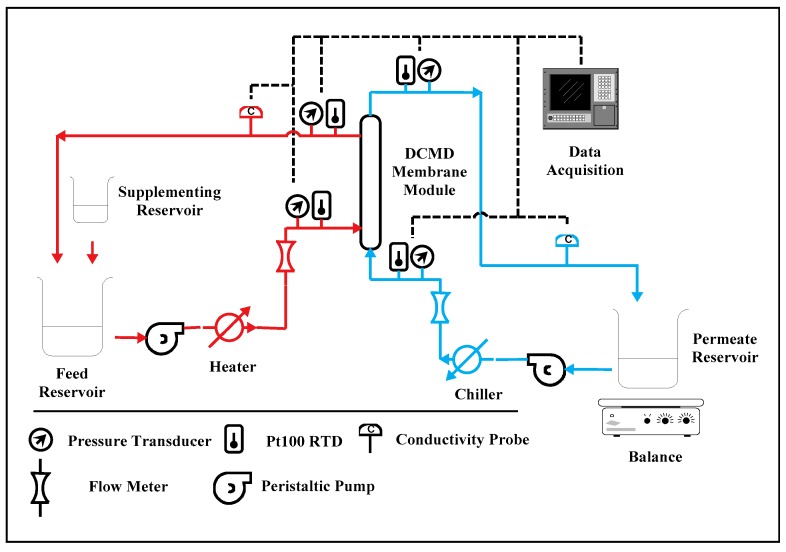
The lab-scale experimental setup for direct contact membrane distillation (DCMD).

For the lab-scale membrane performance testing, all modules were evaluated in a co-current DCMD configuration with the experimental setup shown in Figure 1. Membrane water flux and the associated salt rejection were quantified at various average water-vapor pressure gradients (VPG). Water flux, salt rejection, and the average water-vapor pressure gradient were defined in Equations (2)–(4).

(2) $$Water\;Flux\;\left(\frac{liter}{m^{2}-hr},\;LMH\right)=\frac{\Delta V}{A\cdot \Delta t}$$

(3) $$Salt\;Rejection\;\left(\%\right)=\left(1-\frac{C_{p}}{C_{f}}\right)\times 100\%$$

(4) $$Average\;Vapor\;Pressure\;Gradient\;\left(\frac{MPa}{cm}\right)=\frac{\left(\frac{P_{f,\;i}-P_{p,\;i}}{\delta }+\frac{P_{f,o}-P_{p,o}}{\delta }\right)}{2}$$

where ΔV is the volume of the permeate, A is the membrane surface area per module, and Δt is the measurement time interval. $C_{p}$ and $C_{f}$ are the TDS concentrations in the permeate and feed, respectively. $P_{f,i},P_{f,o},P_{p,i},\;P_{p,o}$ are the water-vapor pressures at the feed inlet and outlet, and those at the permeate inlet and outlet. δ is the membrane thickness. Water-vapor pressure gradient, instead of temperature gradient, across a membrane is a better process parameter for DCMD since it is scaled linearly with water flux. Temperature gradient, on the other hand, is scaled non-linearly with water flux due to the exponential increase of water vapor pressure with temperature and consequently, the same temperature gradient may represent different driving forces for water diffusion [43].

The main objective of the lab-scale study was to quantify water flux and the associated rate of water production per module for the PTFE and PVDF HFMs. The impact of packing density and temperature decline along the module on membrane performance was also investigated. Three packing densities, including 10%, 25%, and 50%, were employed for PTFE and PVDF modules. For all the testing, the feed-side and permeate-side fluid velocities were maintained at 0.06 m/s and 0.2 m/s respectively, regardless of the fiber packing density. The intent was to maintain the same level of temperature polarization at the membrane surfaces for data comparison. The temperature polarization (TP) is defined as the temperature difference between the bulk stream and the membrane surface, and is a strong function of the fluid velocity [44]. Severe TP can limit the availability of thermal energy in the hot feed for membrane distillation, leading to reduced water flux. For the effect of temperature decline along the module length, the water-flux values generated for PVDF modules with effective fiber lengths of 9 cm and 24 cm were compared. A minimum of 3 membrane modules per packing density were evaluated to account for variance in operating conditions and fabrication consistency. Sodium chloride solution with a TDS concentration of 5000 mg/L was used as the feed for the modules. To maintain a constant feed concentration as permeate was generated, de-ionized water was dripped from a supplementing reservoir into the feed reservoir to make up the loss. Hot brackish water was run on the feed (shell) side of the membrane while cool, purified, water flowed through the lumen. The cold stream was pumped through a stainless-steel heat exchanger with a peristaltic pump (model: 77800-60, Cole-Parmer, Vernon Hills, IL, USA) before entering the permeate (lumen) side of a module. The hot stream was pumped through the shell-side of a module with a rotary piston pump (model Q, FMI, Syosset, NY, USA) and the feed temperature was controlled using a hot water bath (model: 2335, Fisher Scientific, Hampton, NH, USA). Flow rates through a module were monitored with rotameters (Cole-Parmer, model T-03219-31). Condensate generated in the lumen was collected in a permeate reservoir, then recirculated into the module. The permeate reservoir was placed on a balance and cumulative mass was measured over time to estimate water flux. Temperature and mass were measured and recorded at 5-min intervals for the duration of the test. Water samples from the feed and permeate were taken periodically to measure the conductivity of both streams with conductivity cells (Cole-Parmer, *K* = 10 and 0.1, 10 kΩ ATC). For each VPG, the test was run for at least 45 h after an equilibration period.

### 2.4. Field Testing

Field testing of the DCMD system for desalination was conducted at Masson Farms with geothermal brackish groundwater. Masson Farms, located in Radium Springs New Mexico, is the second largest geothermally heated industrial greenhouse in the United States [13]. A well descending about 240 m is used to extract geothermal water from a highly fractured rhyolite dike for space heating of the greenhouse at a flow rate of about 6540 m^3^/day. Temperature and TDS of the geothermal water are 92 °C and 3800 mg/L, respectively. The geothermal water is circulated through plate and frame heat exchangers that transfer heat from the geothermal stream to a recirculating freshwater stream for space heating. The geothermal water has a temperature of about 70 °C after the heat exchangers and is then pumped back into the rhyolite dike reservoir downstream. Many of the plant species grown at the greenhouse are extremely sensitive to the salt concentration in water. Therefore, water from permitted freshwater wells and the Rio Grande River must be treated to reduce TDS from 1800 to 300–400 mg/L before it may be used for irrigation. Currently, the greenhouse relies on reverse osmosis (RO) to produce 872 m^3^/day of permeate. Chemical treatment is applied to the feed water for inorganic fouling control, which increases the energy footprint and operating cost of the greenhouse. Desalination of the brackish groundwater using DCMD for irrigation was evaluated at the site for cost reduction. Innate heat in the post-heat-exchanger geothermal fluid was used as the energy source for the DCMD process. Composition of the geothermal brackish groundwater is listed in Table 1 [13].

#### Field DCMD System

**Figure 2:**
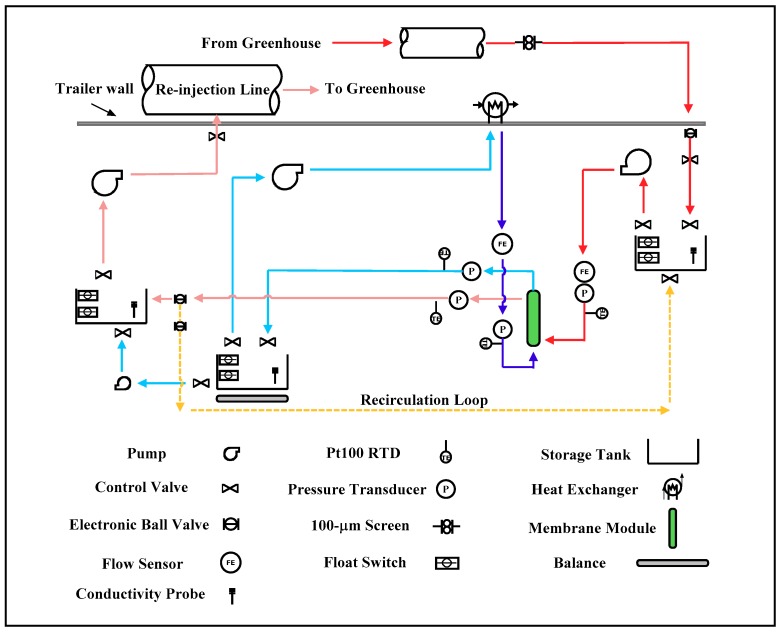
Piping and instrumentation diagram of the field DCMD system.

A mobile DCMD system was constructed and housed in a 6-m trailer for field testing. The geothermal raw water under the line pressure at the facility was filtered through a coarse (~100 µm) screen filter to remove potential particulates. The pressurized water then filled the source-water tank for usage. The filling was controlled by a pair of float switches coupled with a motorized ball valve at the inlet of the tank. A conductivity probe was mounted inside the tank to monitor TDS of the raw water. For DCMD experiments, the raw water was pumped from the tank using a centrifugal pump controlled by a variable frequency drive (VFD) into fabricated hollow-fiber membrane modules. There were four sensor banks located at the shell-side and lumen-side inlets as well as outlets of the module array. Each sensor bank included a pressure transducer and a RTD (Pt100) temperature sensor, with the exception of the outlet banks, which also included conductivity probes for TDS measurements. The post-module concentrate entered a hot-water holding tank from where the fluid was injected via a centrifugal pump into the facility’s process line downstream of the source water intake. A check valve was installed between the concentrate discharge pump and the facility’s process line to prevent backflow. The permeate generated flowed directly into a clean-water holding tank where the water accumulated over time was monitored by weight (balance) or water level (ultrasonic level sensor). A centrifugal pump took the warmed water from the clean-water holding tank and circulated it through an air-cooled radiator to reduce the water temperature before returning it back to the modules for membrane distillation. Periodically, the accumulated water in the clean-water tank was pumped to the hot-water tank for disposal. For geothermally-heated horticulture operations, pumping of geothermal fluid for space heating will transition from a continuous mode to an intermittent mode as the weather gets warmer. A hot-water recirculation loop through the membrane module array was incorporated in the design to minimize frequent system shutdown during this period, which often led to equipment failure. The feed and permeate flow rates were monitored via either infrared-based turbine sensors or ultrasonic flow sensors. A piping and instrumentation diagram for the field DCMD system is provided in Figure 2.

## 3. Results and Discussion

### 3.1. Membrane Characteristics

**Figure 3:**
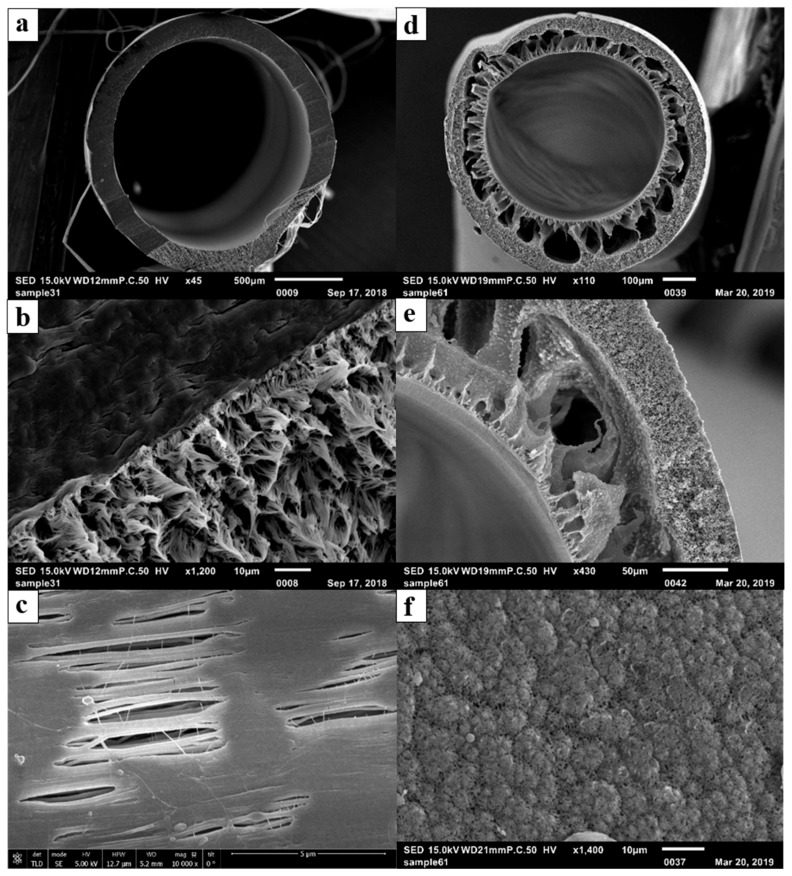
Scanning electron microscopy (SEM) images of the hollow fiber membranes (**a**) polytetrafluoroethylene (PTFE) fiber, (**b**) PTFE cross-section, (**c**) PTFE external surface, (**d**) polyvinylidene fluoride (PVDF) fiber, (**e**) PVDF cross-section, and (**f**) PVDF external surface.

The fabricated PVDF membrane possesses an asymmetric configuration, consisting of an external sponge layer and an internal macro-void layer as shown in Figure 3. During DCMD, hot brackish feed was in contact with the exterior (shell-side) of the HFMs while cold distilled water was in contact with the interior (lumen-side) of the HFMs. The intention was to use the tight pore structure of the sponge layer to prevent the brackish water from intruding into the membranes and low tortuosity of the macro-void layer to facilitate water-vapor transport through the membranes. The PTFE membrane has a rather symmetric pore structure as shown in Figure 3. The uni-axial tension applied during the fabrication process created elliptically shaped pores on the external surfaces of the membranes.

Table 2 shows the characteristics of the hollow fiber membranes. The wall thickness of the PTFE membrane is about 1.5 times thicker than that of the PVDF membrane and the associated porosity is about 5/8 of that for the PVDF membrane. Therefore, it was anticipated that the PVDF membrane would produce a higher water flux than the PTFE membrane for a similar pair of inlet temperature in the feed and permeate to the lab-scale DCMD system. The PTFE membrane has a nominal pore size about 1.5 times of that for the PVDF membrane and also possesses a much wider pore-size distribution. This is likely the reason why the values of liquid entry pressure for water (LEP_w_) for the two types of membranes are similar at 22 °C despite the higher hydrophobicity for PTFE. It is important to note that LEP_w_ was observed to decrease with increase of water temperature and can constrain the operation of DCMD systems at elevated temperature [45]. For mechanical properties, the PTFE fiber has a much higher Young’s modulus than the PVDF fiber, suggesting that the former is more brittle and has a higher degree of crystallinity. Its failure stress is also substantially higher than that of the PVDF fiber and can facilitate the module-making process.

### 3.2. Lab-Scale Membrane Performance

**Figure 4:**
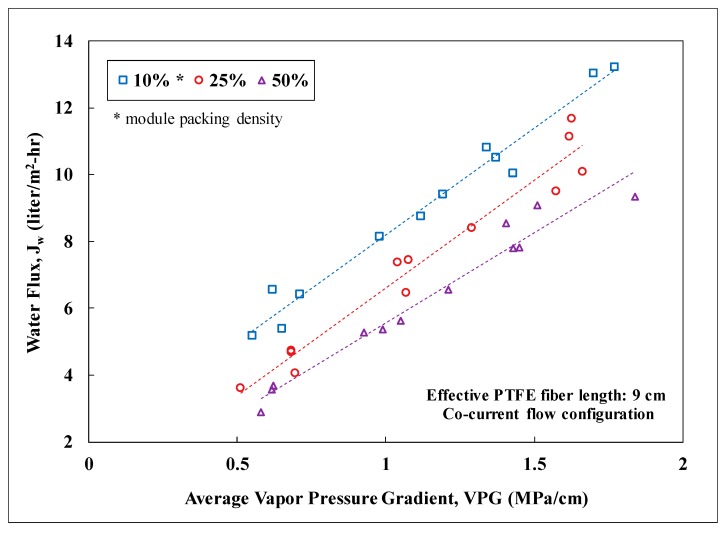
Water flux as a function of the average water-vapor pressure gradient for 10, 25, and 50% module packing densities in PTFE membrane modules. Other experimental conditions: Lab testing, feed: NaCl solution with TDS of 5000 mg/L, effective fiber length: 9 cm, co-current flow, stable water flux for 45 h, salt rejection: over 99%.

**Figure 5:**
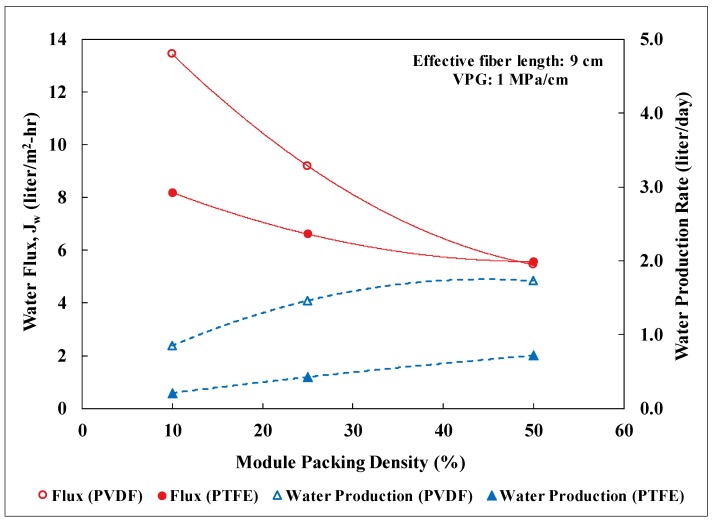
Module performance in water flux and production rate for PTFE and PVDF hollow fiber membranes as a function of packing density at a vapor pressure gradient of 1 MPa/cm. Other experimental conditions: Lab testing, feed: NaCl solution with TDS of 5000 mg/L, effective fiber length: 9 cm, co-current flow, stable water flux for 45 h, salt rejection: over 99%.

Water flux of the membrane modules at packing densities of 10, 25, and 50% was measured as a function of the average water-vapor pressure gradient imposed on the modules. Since the vapor pressure gradient is the driving force for water diffusion across the membranes, it was observed to scale linearly with the water flux for a specific packing density. Figure 4 shows the correlations for PTFE membrane modules with an effective fiber length of 9 cm. The membrane surface area for a 10% packed module was about 0.0011 m^2^ and increased proportionally with packing density. As the packing density increased, the observed water flux declined. This reduction of water flux was probably attributed to the decline of the total heat flow into the module while maintaining the average shell-side fluid velocity. Randomly-packed hollow fibers with an increasing packing density could also result in progressively uneven distribution of fluid flow and thus heat flow among the fibers. A similar trend with packing density was also observed for the PVDF membrane modules. For a 10% packing density, the PVDF-based modules had about 0.00264 m^2^ of membrane surface area per module. Figure 5 presents a performance comparison between the PTFE and PVDF membrane modules at an average vapor pressure gradient of 1 MPa/cm (e.g., hot inlet: 55–60 °C and cold inlet: 22–25 °C). For low packing density (i.e., 10%) when the thermal energy for distillation is abundant, the PVDF modules exhibited a significantly higher water flux (~1.6 times) than the PTFE module due to the larger membrane surface area per module. As the packing density increased from 10 to 50%, similar water flux, however, was observed for both types of modules while the ratio of the membrane surface area between PVDF and PTFE remained about the same at 2.5. This convergence of observed water flux at high packing density was likely in part due to the lack of thermal energy for distillation as the total membrane surface area in a module grew. Nevertheless, the module water production rate for the PVDF membranes was still 2.5 times of that for the PTFE membranes at 50% packing density. This result signifies the importance of reducing fiber diameter in maximizing the water production rate per module for DCMD. The water flux values observed in this study for PVDF and PTFE HFMs were also compared to those reported in the literature for PTFE membranes as shown in Table 3 [38,46,47]. Water flux of the fabricated PVDF HFM in this study is comparable to those reported for commercial PTFE flat-sheet membranes under similar inlet temperatures. The water flux values observed for the PTFE HFM tested in this study are higher than that reported in the literature for a PTFE HFM. However, depending on the module packing density, these values were about 15% to 68% lower than those reported for commercial PTFE flat-sheet membranes. It is important to note that the active-layer thickness for the PTFE HFM in this study is 2.7 to 6 times thicker than those for the commercial PTFE flat-sheet membranes and therfore generated substantially higher resistance for mass transfer of water vapor.

**Figure 6:**
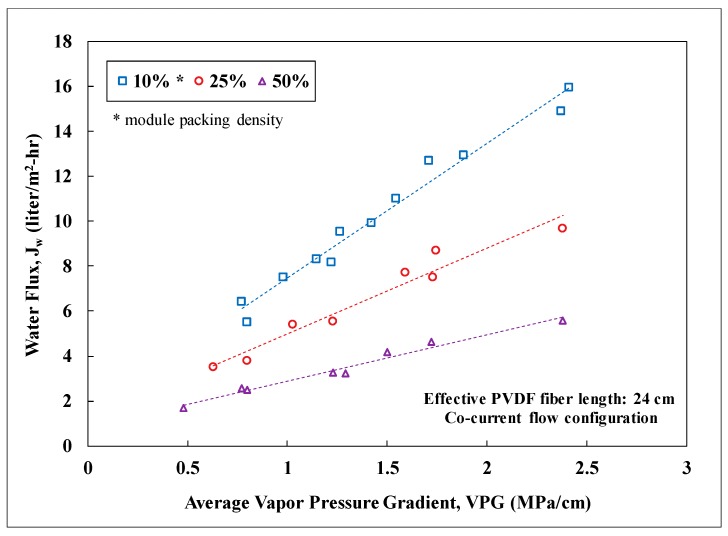
Water flux as a function of the average water-vapor pressure gradient for 10, 25, and 50% module packing densities in PVDF membrane modules. Other experimental conditions: Lab testing, feed: NaCl solution with TDS of 5000 mg/L, effective fiber length: 24 cm, co-current flow, stable water flux for 45 h, salt rejection: over 99%.

Water flux for PVDF membrane modules with an effective fiber length of 24 cm was also recorded for different packing densities as shown in Figure 6. The results for the long and short (i.e., 24 cm vs. 9 cm) PVDF membrane modules were compared to examine the impact of temperature decline along the module length. As expected, increasing module length led to lower water flux at a constant packing density. For the long modules, severe temperature decline along the module length as a result of insufficient heat flow diminished the rate of water distillation for membrane area closer to the module outlet, leading to a lower average water flux for the module. At 50% packing density, the long module provided about 53% of the water flux for the short module and yet delivered 42% more in the module water production rate due to 2.7 times of membrane surface area. For the long modules, the water flux and associated production rate can be further boosted by increasing the fluid flow rate on the shell side of the modules at the expense of higher energy costs for pumping. Salt rejection was recorded for all the experiments and was always greater than 99%.

### 3.3. Field Performance

**Figure 7:**
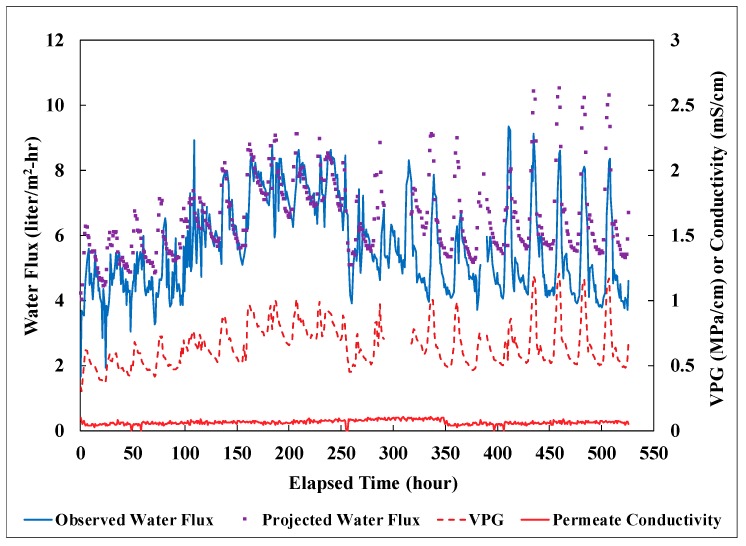
Observed and projected water flux with the associated vapor pressure gradient (VPG) over time for the PVDF membrane modules tested in the field. Other experimental conditions: Field testing, feed: geothermal groundwater with TDS of 3800 mg/L, effective fiber length: 24 cm, 10% packing density, counter-current flow, water flux observed for 530 h, salt rejection: 99%.

**Figure 8:**
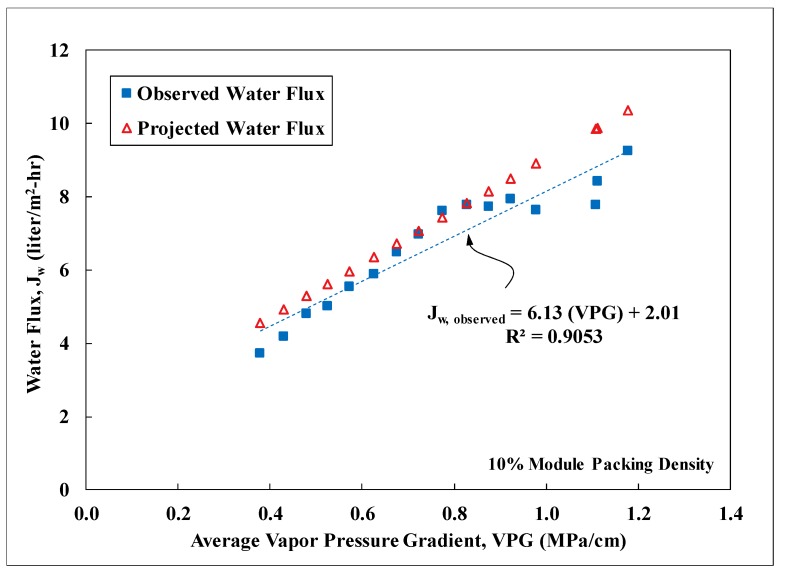
Comparison between the observed and predicted water flux as a function of the average vapor pressure gradient for the field-tested PVDF membrane modules. Other experimental conditions: Field testing, feed: geothermal groundwater with TDS of 3800 mg/L, effective fiber length: 24 cm, 10% packing density, counter-current flow, water flux observed for 530 h, salt rejection: 99%.

PVDF membrane modules fabricated with an effective fiber length of 24 cm and a 10% packing density were tested at Masson Farms for their effectiveness of desalination using the field DCMD system. The geothermal brackish groundwater on site was conveyed to the field system post heat exchangers for water desalination. Temperature of the groundwater at the inlet of the DCMD system fluctuated between 35 to 75 °C with most of the values concentrating within 50 to 60 °C. Temperature of the recirculating permeate ranged from 10 to 33 °C during the field testing. Performance of the modules was monitored for more than 22 days and was shown in Figure 7. Water flux was observed to track closely with the water-vapor pressure gradient imposed on the modules. The salt rejection was consistently about 99% throughout the period. The significant variability of the observed water-vapor pressure gradient was resulted mainly from the temperature fluctuation of the feed into the DCMD system. The flexible hose connecting the facility process-water pipe to the inlet of the DCMD system was not insulated and consequently subjected to the influence of the atmospheric temperature. The projected water flux was calculated based on the lab-scale correlation presented in Figure 6 for modules of a similar size and with a 10% packing density. However, the DCMD process employed during the field testing was in a counter-current flow configuration. This configuration was shown to exhibit higher water fluxes than the co-current configuration used to establish the lab-scale correlation. Therefore, a multiplier of 1.21, obtained from prior studies, was applied to the lab-scale correlation to account for the increase of water flux under counter-current flow configuration [18]. A comparison of the water flux data in Figure 8 showed that the membrane performance in water production did not deteriorate over time. The observed water flux, however, was consistently lower than the projected water flux. In order to estimate the average percentage of reduction, the observed water-flux data was sorted in a descending order and separated into different groups based on the associated average vapor pressure gradient (VPG). The water flux data in each group with a VPG span of 0.05 MPa/cm, was then averaged. The results shown in Figure 8, indicated an average reduction of 10% in water flux when compared to the corrected lab-scale correlation. The reduction was probably caused by the particulate foulants from the corrosion of cast-iron pipes in the facility and the magnitude of reduction was consistent with the data observed during lab-scale fouling testing [18].

During the field testing, deposition of reddish particulate matter on membrane surfaces was observed over time. It was evidenced that the 100-μm pre-filter at the inlet of the field DCMD system was not sufficient to capture all the particulate matter. In order to determine the nature and surface loading of the particulate matter, the hollow fiber membranes were submerged in de-ionized water and sonicated to dislodge the particulate matter at the end of the field testing. The resulted water was then filtered with a 5-μm filter, which was subsequently dried at 70 °C for an hour before being weighed. Based on the results, the surface loading was estimated to be about 300 mg per m^2^ of membrane surface area. The dried particulate matter was analyzed using X-ray diffraction (XRD) and was determined to be mainly iron oxides, probably a corrosion product of the cast-iron pipes used at Masson Farms.

Based on the field experience, implication for system design and operation was provided for field DCMD systems with PVDF-based HFMs that utilize geothermal brackish groundwater as the source. From the system design perspective, HFM modules should preferrably be oriented in an upright orientation with both the feed and permeate flowing co-currently upward. Gas evolution is common for geothermal groundwater and can create gas lock in improper module setup to obstruct flow through the modules. A recirculation loop should be incorporated in the system design to minimize membrane fouling and equipment failure during the warmer seasons when geothermal groundwater is available intermittently for space heating. Chilling permeate to increase the vapor pressure gradient for water production can be energy intensive. Heat exchnagers using air cooling are the primary candidates based on economic considerations. For the field operation, the hightest frequency of system failure occurred during startup and shutdown when the hydraulic pressure differential across the membranes fluctuated the most. Consequently, pressure monitoring and proper selection of pumping systems are needed to minimize the risk. Generally, groundwater with higher temperature is chosen as the source for DCMD systems to increase the water flux. However, LEP_w_ can be significantly reduced with the increase of water temperature. For example, the LEP_w_ of the fabricated PVDF HFMs in this study was reduced by 60% from 1.32 to 0.53 bar as the water temperature increased from 22 to 81 °C. This reduction of LEP_w_ increases the risk of brackish water intrusion and subsequent loss of water flux during the system startup and shutdown.

## 4. Conclusions

PVDF hollow fiber membranes (HFMs) were fabricated and compared to commercially available PTFE HFMs in their effectiveness for desalination using DCMD. The PVDF HFMs exhibited much higher water flux than the PTFE HFMs at 10% packing density, resulting mainly from thinner membrane wall and higher porosity. Both types of HFMs, however, showed similar water flux at 50% packing density possibly due to insufficient thermal energy in the feed. Relative to commercial PTFE flat-sheet membranes, the PTFE HFMs possessed water flux that was about 15% to 68% lower, depending on the packing density. The deficiency can most likely be rectified by reducing the active-layer thickness of the PTFE HFMs, which is about 2.7 to 6 times thicker than those for the commercial PTFE flat-sheet membranes. Increasing module packing density and length had a negative impact on water flux. For example, as the packing density of the short PVDF modules increased from 10 to 50%, the water flux decreased by 60% from 13.4 to 5.4 LMH at an average vapor pressure gradient of 1 MPa/cm. Despite the declining of water flux, the water production rate per module increased gradually with the increase of total membrane surface area from raised packing density or module length. The results affirmed the needs to reduce fiber diameter, maximize packing density, and increase module length for water production. It is anticipated that increasing the feed flow rate would further boost the water flux, however, at the expense of a higher pumping cost. Membrane performance of PVDF modules with an effective fiber length of 24 cm and a 10% packing density was also evaluated at the 2nd largest, geothermally-heated, commercial greenhouse in the United States with on-site geothermal brackish groundwater. The water flux and salt rejection observed were fairly stable over more than 22 days in the presence of particulate foulants and severe temperature swing. The results demonstrated the robustness of the DCMD system in the field.

## Figures and Tables

**Table 1:** Composition of the geothermal brackish groundwater *.

Parameter	Masson Farms
Ca^+^	104
Mg^2+^	11.4
K^+^	191
Na^+^	1221
Li^+^	1.1
Fe^2+^	0.2
Sr^2+^	2.3
F^−^	5.5
Cl^−^	2022
${\mathrm{SO}}_{4}^{2-}$	27.6
${\mathrm{HCO}}_{3}^{-}$	11.6
B	0.9
Si (as SiO_2_)	58.2
pH	7.5
TDS	3800
Temperature (°C)	92

* Units are in mg/L unless otherwise noted.

**Table 2:** Characteristics of the PTFE and PVDF Hollow Fiber Membranes.

Membrane Characteristic		PVDF	PTFE
Outer diameter (µm) ^a^		841 ± 5	1799 ± 50
Wall thickness (µm) ^a^		122 ± 22	178 ± 12
Macrovoid to sponge ratio ^b^		1.08	N/A
Pore size		0.319/0.333/0.422 ^c^	0.385/0.495/0.831 ^d^
Porosity		0.79 ± 0.05	0.50 ± 0.04
Failure stress		1.32	>21.5
Young’s modulus		15.66	348
Liquid entry pressure, LEP_w_ (bar)	at 22 °C	1.32	1.37
	at 81 °C	0.53	-

^a^ nominal size based on post-processing of SEM images. ^b^ ratio of the macrovoid-layer thickness to the sponge-layer thickness. ^c^ minimum pore size/mean pore size/maximum pore size. ^d^ based on the short axis of the elliptical pore.

**Table 3:** Comparisons of Properties and Performance for Selected PVDF and PTFE MD Membranes.

Membrane Characteristic		This Study		This Study		Zhang et al. ^a^	Millipore ^b^	Membrane Solutions ^c^	GE Osmonics ^c^
Type		Hollow fiber		Hollow fiber		Hollow fiber	Flat sheet	Flat sheet	Flat sheet
Material		PTFE		PVDF		PTFE	PTFE	PTFE	PTFE
Membrane configuration		Symmetric		Sponge/macrovoid		Symmetric	N/A	Active layer/fabric	Active layer/scrim
Thickness	Active	178		122		365	N/A	30	67
	Support	N/A		N/A		N/A	N/A	185	97
Nominal pore size of active layer (µm)		0.5		0.3		0.3	0.25	1	0.45
Porosity (active layer)		0.5		0.79		0.85	0.7	0.92	0.88
Feed TDS (mg/L)		5000		5000		10,000	29,250	10,000	10,000
Flow configuration		Co-current		Co-current		Counter-current	Counter-current	Counter-current	Counter-current
Module packing density (%)		10	50	10	50	N/A	N/A	N/A	N/A
Feed inlet (°C)		65	64	62	64	60	65	60	60
Permeate inlet (°C)		22	31	28	36	20	15	20	20
Water flux (LMH) ^d^		9.4	5.3	17.3	5.7	4	12.6	11	16.5

^a^ based on reference [38]. ^b^ FGLP 1425, based on reference [45]. ^c^ based on reference [46]. ^d^ LMH = liters per m^2^ of membrane area, per hour.
